# IL-1 Family Members Mediate Cell Death, Inflammation and Angiogenesis in Retinal Degenerative Diseases

**DOI:** 10.3389/fimmu.2019.01618

**Published:** 2019-07-16

**Authors:** Yvette Wooff, Si Ming Man, Riemke Aggio-Bruce, Riccardo Natoli, Nilisha Fernando

**Affiliations:** ^1^The John Curtin School of Medical Research, The Australian National University, Canberra, ACT, Australia; ^2^ANU Medical School, The Australian National University, Canberra, ACT, Australia

**Keywords:** retinal degeneration, IL-1 (interleukin-1), age-related macular degeneration (AMD), inflammation, cytokine, inflammasome, IL-1b, photoreceptor cell death

## Abstract

Inflammation underpins and contributes to the pathogenesis of many retinal degenerative diseases. The recruitment and activation of both resident microglia and recruited macrophages, as well as the production of cytokines, are key contributing factors for progressive cell death in these diseases. In particular, the interleukin 1 (IL-1) family consisting of both pro- and anti-inflammatory cytokines has been shown to be pivotal in the mediation of innate immunity and contribute directly to a number of retinal degenerations, including Age-Related Macular Degeneration (AMD), diabetic retinopathy, retinitis pigmentosa, glaucoma, and retinopathy of prematurity (ROP). In this review, we will discuss the role of IL-1 family members and inflammasome signaling in retinal degenerative diseases, piecing together their contribution to retinal disease pathology, and identifying areas of research expansion required to further elucidate their function in the retina.

## IL-1 Family Members

### Introduction to the IL-1 Family

The interleukin-1 (IL-1) family is a central mediator of innate immunity and inflammation [reviewed by Dinarello (1)]. IL-1 family members have been widely associated with both the development and progression of inflammatory diseases, and in particular have been linked to neurodegenerative and neuroinflammatory diseases such as Alzheimer's disease (2–5), stroke (6), cerebral ischemic cell death (7), Multiple Sclerosis (8, 9), Parkinson's disease (10, 11), Down syndrome (3), and retinal degenerative diseases including Age-Related Macular Degeneration (AMD).

The IL-1 family of cytokines has 11 members, which are further subdivided into three groups; the IL-1, IL-18, and IL-36 subfamilies. The IL-1 cytokine subfamily includes agonists (IL-1α, IL-1β, and IL-33) as well as receptor antagonist, IL-1Ra; the IL-18 subfamily comprises agonists IL-18 and IL-37, and the IL-36 subfamily is made up of agonists IL-36α, β, γ, and receptor antagonists IL-36Ra and IL-38 (1). In addition, there are 10 members of the IL-1 receptor (IL-1R) family which are able to bind specific IL-1 ligands in combination with a co-receptor, and perform pro- and anti-inflammatory functions (1).

IL-1β, IL-18, and IL-1α are the most widely researched IL-1 family members associated with retinal degenerative diseases (Table 1), having pro-inflammatory actions, and in the case of IL-18, a role in angiogenesis (49, 50). IL-1β and IL-1α are known to exert similar biological effects (51), acting on IL-1R, eliciting pro-inflammatory actions following activation. However, unlike IL-1α which is both constitutively expressed and active in its 31 kDa pro-form, IL-1β is only produced in its inactive 35 kDa pro-form following priming signals, such as pathogen- or damage-associated molecular patterns (PAMPs or DAMPs), and is only subsequently cleaved to its 17 kDa active form following inflammasome activation in damaged or diseased states (1, 2, 52–54). While IL-1α is suggested to act early in inflammation by inducing neutrophil immune cell recruitment, IL-1β is thought to act in the later phase of macrophage recruitment to damaged tissue (51).

**Table 1:** Tissue-specific expression of IL-1 family members associated with retinal degenerative diseases.

**Name**	**Receptor**	**Mechanism**	**Retinal Degeneration**	**References**	**Tissue**	**Tags per million (TPM)**	**Confidence (*z*-score)**	**Enhanced expression (TPM)**
IL-1α	IL-1RI	Pro-inflammatory	Dry AMD DR Glaucoma	(12, 13) (14) (15)	Retina Brain	0 1.1	1 (1.1) 3 (4.4)	Tonsils (22)
IL-1β	IL-1RI	Pro-inflammatory	Dry AMD Wet AMD Glaucoma RP ROP DR	(16–18) (12, 19, 20) (21) (22, 22, 23) (24–26) (27–29)	Retina Brain	3.8 9.7	3 (4.4) 4 (7)	Spleen (78)
IL-18	IL-18Rα	Pro-inflammatory	Wet AMD Dry AMD RP ROP Glaucoma	(30–33) (34–37) (23) (38–40) (41)	Retina Brain	12 12	2 (2.6) 3 (4.1)	*Expressed in all* Esophagus (167)
IL-1Ra	IL-1RI	Antagonist for IL-1α, IL-1β	DR ROP	(27, 28, 42, 43) (39, 44)	Retina Brain	1.7 0.8	N/A 3 (3.8)	Tonsils (1553)
IL-33	ST2	Pro-inflammatory	Dry AMD Wet AMD	(45) (46)	Retina Brain	N/A N/A	N/A N/A	N/AN/A
IL-36α	IL-1Rrp2	Pro-inflammatory	N/A	N/A	Retina Brain	0 0	N/A N/A	Tonsils (391)
IL-36β	IL-1Rrp2	Pro-inflammatory	N/A	N/A	Retina Brain	N/A N/A	N/A N/A	N/A N/A
IL-36γ	IL-1Rrp2	Pro-inflammatory	N/A	N/A	Retina Brain	N/A N/A	N/A N/A	Tonsils (24)
IL-36Ra	IL-1Rrp2	Antagonist for IL-36α, IL-36β, IL-36γ	N/A	N/A	Retina Brain	0 0	N/A N/A	Tonsils (25)
IL-37	Unknown	Anti-inflammatory	DR	(47)	Retina Brain	N/A N/A	N/A N/A	N/A N/A
IL-38	IL-1Rrp2	Anti-inflammatory	ROP	(48)	Retina Brain	N/A N/A	N/A N/A	N/A N/A

*Tags Per Million (TPM) provided from the human protein ATLAS, provided by FANTOM5 consortium as part of the human protein Atlas (https://www.proteinatlas.org/). Confidence scores provided from the Tissues Expression database (https://tissues.jensenlab.org/About). Main RNA tissue category (Enhanced Expression) is shown in TPM. “Expressed in all” means all tissues investigated produced that molecule (IL-18)*.

Conversely, IL-18, which acts on the IL-18Rα/β receptor, is both constitutively expressed in its pro-form, but cleaved into its active form following inflammasome activation (51). Interestingly, IL-18 has been reported to have both anti- and pro-inflammatory actions, but is also more widely known for its angiogenic roles (49, 50). In addition to known pro-inflammatory activities, IL-1 family members can also participate in anti-inflammatory pathways, with certain IL-1 family members (IL-33 and IL-1α) having dual functions, being able to bind to DNA or the cell membrane receptor and elicit differential effects (1, 46, 55, 56).

In this review, we will discuss the role of IL-1 family members in retinal degenerative diseases, piecing together their contribution to retinal disease pathology, and identifying areas of research expansion required to further elucidate their function in the retina. Furthermore, we will elaborate on some of the mechanisms of IL-1β activity in degeneration, the most highly studied IL-1 family member in the retina.

### Inflammation in Retinal Degenerative Diseases

The retina is part of the central nervous system (CNS) and is a specialized sensory tissue lining the posterior surface of the eye. Photoreceptors, specialized light-sensing retinal cells, have the ability to convert light into electrical signals, which are transmitted to the brain via the optic nerve. Both inherited and acquired retinal degenerative diseases can occur when retinal homeostasis is disrupted. This is caused by a combination of genetic mutations (57), the accumulation of reactive oxygen species (ROS) (58), and inflammation in aging (59, 60). The progression of both inherited and acquired retinal degenerative diseases share several features in common, including chronic increases in both oxidative stress and inflammation (59, 61, 62). Increased activation, migration, and recruitment of resident microglia and blood-borne macrophages are characteristic of progressive photoreceptor degeneration in AMD (16, 17, 63–66), diabetic retinopathy (67, 68), retinitis pigmentosa (69–72), glaucoma (73–75), and retinopathy of prematurity (44).

Microglia and macrophages are the primary leukocyte populations found in the retina during disease, and one critical mechanism by which these cells cause damage in retinal degenerations is through activation of the inflammasome. The inflammasome is an oligomer protein complex that leads to the maturation and secretion of two IL-1 family members, IL-1β and IL-18, into the extracellular environment (76). The assembly and activation of the NOD-like receptor pyrin domain-containing 3 (NLRP3) inflammasome, the most well-characterized inflammasome, is stimulated by several mechanisms, including Toll-like receptor (TLR) signaling and purinergic receptor signaling (76), the latter which is activated by extracellular ATP released by dying cells (76, 77). The migration and recruitment of microglia and macrophages is associated with an increased production of chemokines and cytokines, including IL-1β, as well as complement activation, which leads to progressive photoreceptor degeneration [reviewed in Ambati et al. (78) and McMurtrey and Tso (79)].

## Age-Related Macular Degeneration

### AMD Disease Pathogenesis

AMD is the leading cause of irreversible blindness in the Western World, primarily affecting the aging population. The estimated prevalence is expected to be 288 million worldwide by 2040, posing a significant global economic burden (80). Although neovascular “wet” AMD currently is treated using anti-vascular endothelial growth factor (VEGF) intravitreal injections to prevent choroidal neovascularisation (CNV) (81, 82), early “dry” AMD and late-stage atrophic dry AMD are currently untreatable. In dry AMD, there is a gradual loss of retinal pigment epithelium (RPE) cells and photoreceptors in the outer retina, leading to the development of a retinal lesion in the specialized macular region, responsible for central high visual acuity, which progressively expands over time (83). The cumulative loss of outer retinal cells and the expansion of the atrophic lesion results in a large drop in visual function in patients with the disease (84). Development of a 2 mm lesion in the foveal region within the macula can result in legal blindness (85).

Immune-based therapies are being explored as the most likely drug candidates for clinical trials, due to the significance of immunological processes in the pathogenesis of AMD. This includes a number of complement system inhibitors to control this major inflammatory pathway such as APL-2 (Apellis Pharmaceuticals) for the treatment of advanced dry AMD (78, 86). Dysregulation of the immune system is critically linked to the development of advanced dry AMD, including the recruitment and activation of resident microglia to the outer retina, and the persistent accumulation of subretinal macrophages recruited from the vasculature, which together are the primary immune cells of the retina under damage conditions (64, 66, 87–91). It has been demonstrated that retinal microglia and macrophages are centrally involved in AMD pathogenesis, including production of various innate immune system components such as complement (65), chemokines (92), and IL-1β (18). This suggests that these microglia and macrophages are major therapeutic targets for the treatment of dry AMD, in which progressive atrophic lesion expansion is promoted by activation of these cells.

### IL-1α as a Potential Initiator of the Inflammasome in AMD

The release of interleukin-1α (IL-1α), a 31 kDa constitutively expressed member of the IL-1 family, is known to be both inflammasome-dependent and independent (93, 94). However, in a positive feedback loop, IL-1α is also known to prime the assembly of the NLRP3 inflammasome in the retina, with inflammasome priming using IL-1α in RPE cells increasing the damage caused by blue light-induced oxidative stress (95). In this model, the accumulation of lipofuscin, lipid-containing pigment granules that build up during aging, occurs in the photoreceptor outer segments causing oxidative damage (95). IL-1α stimulation altered the cell death profile of damaged RPE cells from apoptosis to pyroptosis, an inflammatory cell death pathway dependent on inflammasome activation (95). Other studies have indicated that IL-1α is a danger signal, or “alarmin,” released from stressed or dying RPE cells, leading to the secretion of other pro-inflammatory cytokines from these cells (96, 97). As RPE cell death is central to the progression of AMD, IL-1α has been suggested as a therapeutic target for controlling sterile retinal inflammation. In AMD patients, serum levels of IL-1α amongst other cytokines are significantly higher compared to healthy control patients (12), a trend which was also observed in plasma in a rat model of ischemia/reperfusion injury (13). These data indicate that IL-1α could be biomarker for retinal diseases.

### The Role of IL-1β in AMD

Interleukin-1β (IL-1β) is a pro-inflammatory cytokine produced as a 35-kDa precursor, however following inflammasome activation is cleaved by protease enzyme Caspase-1 (CASP1) into a 17-kDa active form (51, 98, 99). Active IL-1β has known roles in initiating and propagating sterile inflammation, including macrophage recruitment (100), activation of the pro-inflammatory cytokine interleukin-6 (IL-6) (101) and modulating chemokine expression (18), which in retinal degenerative diseases such as AMD are characteristic pathogenic features that ultimately result in progressive photoreceptor cell death. Although inflammasome signaling is thought to play both protective and detrimental roles in wet and dry AMD due to the production of IL-18 (30, 31, 34, 102–106), the synthesis of mature IL-1β is well-established in the pathogenesis of both forms of AMD (19, 107). IL-1β dysfunction has been associated with excessive inflammation in retinal degenerations using animal models (16–18, 20, 70, 108, 109), including those modeling key features of dry AMD.

However, the mechanisms behind inflammasome-mediated production of active IL-1β and the subsequent induction of photoreceptor cell death and retinal damage is unclear. Although IL-1β has not been conclusively linked to dry AMD in human patients (103, 110), the role of IL-1β in AMD pathogenesis has been investigated both *in vivo* in rodent dry AMD models, as well as *in vitro*. An increase in IL-1β was found in the vitreous of rats injected intravitreally with drusen component amyloid beta (Aβ) (111, 112), a toxic peptide aggregate known to accumulate in neurodegenerative diseases such as Alzheimer's disease [reviewed in Murphy and LeVine (113)], as well as in dry AMD (114, 115). In photo-oxidative damage models that mimic several facets of dry AMD pathogenesis (116), it has been found that the gene and protein expression of IL-1β was up-regulated in the photo-oxidative damaged rodent retina (18, 108, 117–119). It was demonstrated that inhibition of IL-1β using both small interfering RNA (siRNA) and a neutralizing antibody was able to ameliorate retinal degeneration, reducing immune cell recruitment to the outer retina, and production of chemokines (*Ccl2, Cxcl1, Cxcl10*) from both Müller glia and RPE cells, ultimately slowing photoreceptor cell death in retinal degeneration (18).

In addition, others have demonstrated that using a photo-oxidative damage model, cone segment degeneration was correlated with an increased infiltration of IL-1β-expressing mononuclear phagocytes (16). Notably, while mononuclear phagocyte accumulation remained, cone degeneration was abolished following IL-1β inhibition (16). Although the mechanism of IL-1β-dependent photoreceptor cell death is unclear, both photoreceptor degeneration and the associated presence of accumulated mononuclear phagocytes has been reported in dry AMD by the same group (16, 17, 63).

In contrast to most recent literature, previous studies have reported a dose dependent effect of IL-1β, with low doses (5 μg/ml) conferring retinal protection and photoreceptor rescue in The Royal College of Surgeons (RCS) rats (120, 121), a strain with inherited retinal degeneration (122). It is possible that a high dose of IL-1β provides an additional priming signal via the IL-1R signaling axis to amplify the expression of inflammasome components, inducing assembly of the inflammasome and perpetuating photoreceptor cell death. Further investigation into mechanisms by which IL-1β causes photoreceptor cell death may shed light on the protective vs. detrimental roles of IL-1β in the progression of dry AMD and in other retinal degenerations where the gradual inflammatory-mediated loss of photoreceptors is a key feature of disease.

We will further detail the localization and pro-inflammatory functions of IL-1β in retinal degenerations later in this review, focusing on its role in dry AMD pathogenesis.

### Role of IL-18 in Angiogenesis and Neovascular AMD

Mature interleukin-18 (IL-18) is a pro-inflammatory cytokine produced through inflammasome activation (98, 99). The role of IL-18 in retinal degenerations including AMD is controversial in the current literature [reviewed in Campbell et al. (49, 50)], with some sources highlighting a detrimental role for IL-18 in the progression of dry AMD (34, 103), whilst there is also evidence of a protective role of IL-18 for wet AMD, in which angiogenesis and neovascularisation contributes to disease progression (30, 31).

The involvement of IL-18 in angiogenesis and neovascularisation has been well-established demonstrating that IL-18 has a role in the formation of blood vessels in the retina (123). It was observed that in early development, IL-18 knockout mice demonstrated abnormal vessel formation and retinal overexpression of angiogenic factors including VEGF (123). A series of studies by Doyle et al. built upon these foundations demonstrating that IL-18 knockout mice had smaller laser-induced CNV volumes than wild type controls (31). Incubation with recombinant IL-18 reduced VEGF expression in immortalized RPE cells (ARPE-19) and brain-derived microvascular endothelial cells (bEnd.3), indicating a mechanism by which smaller CNV volumes in the IL-18 knockout mice may have occurred (31). The follow up studies by Doyle et al. investigated the potential of IL-18 treatments as therapeutics for wet AMD, including in non-human primates, demonstrating its ability to reduce the pathogenic hallmarks of wet AMD (30, 105). A separate study also found that IL-18 displayed protective effects against neovascularisation (32), in which IL-18 levels increased in the eye after treatment with anti-VEGF, and in mice with ischemic/reperfusion (IR) injury, suppression of VEGF caused an increase in IL-18^+^ myeloid cells. Additionally, an injection of IL-18 reduced CNV (32). Another study demonstrated that NLRP3 and IL-1β signaling promoted VEGF-induced CNV formation, and a deficiency in IL-18 had the same effect on CNV lesion development (33). These studies support the findings from Doyle and colleagues in regards to the anti-angiogenic properties of IL-18 (31).

However, in dry AMD, the literature points to a different contribution of IL-18 in retinal degenerations. A study involving dry AMD patients with the complement factor H (CFH) Y402H polymorphism demonstrated that systemic levels of IL-18 were elevated in patients with the at-risk CC variant for the polymorphism, alongside increased systemic IL-1β (35). Ijima et al. also found that serum IL-18 levels in dry AMD patients were higher than age-matched control patients (34). A series of studies using both mouse models and patients with dry AMD demonstrated that *Alu* RNA mediates RPE degeneration through activation of NLRP3 and IL-18 in these cells (36, 103), possibly through activation of Caspase-8 (CASP8) (37). The same group found a critical role for IL-18 in wet AMD (124).

Overall, the literature surrounding the role of IL-18 in AMD indicates a potential dual role of this pro-inflammatory cytokine in modulating retinal damage in neovascular retinal diseases including wet AMD, which may act differently to its involvement in dry AMD pathogenesis. It is possible that local concentrations of IL-18, like that of IL-1β, determine the protective and detrimental effects of this cytokine in the retina. Further investigations are required to elucidate the mechanisms by which IL-18 may have a role in regulating inflammation in retinal degenerations, including through the induction of interferon-gamma (IFN-γ) (125), a cytokine thought to play a role in AMD (126).

### IL-33 and Cytokine Production in AMD

The agonist interleukin-33 (IL-33) is known to have a key role in both innate and adaptive immunity, activating NFκB and MAPK inflammatory signaling pathways and inducing cytokine release [reviewed in Liew et al. (127)]. IL-33 has been shown to be induced upon Aβ stimulation in RPE cells, where it led to a regulation of IL-1β, IL-6, IL-8, and TNFα (128). Other studies have found that IL-33 is upregulated in activated RPE cells in laser-induced CNV, regulating angiogenesis, tissue remodeling and wound healing (46). These studies suggest that IL-33 regulation in the eye may affect critical cytokine signaling pathways involved in retinal degenerations. However, not only RPE cells have been implicated in IL-33 upregulation in retinal damage, with Müller cells also found to produce IL-33 in late-stage dry AMD donor retinas within the lesion area (45). It was shown that IL-33 production by Müller cells was able to induce the expression of other chemokines and cytokines (45), including CCL2, which has been shown to be produced primarily by Müller cells in retinal degenerations to recruit macrophages into the site of damage (129, 130). Together, these studies indicate that IL-33 signaling by RPE and Müller cells may regulate the migration of microglia and the influx of macrophages into the retina following photoreceptor and RPE damage, by influencing cytokine production.

### IL-1Ra in RPE Cells

Interleukin-1 receptor antagonist (IL-1Ra) is an anti-inflammatory competitive receptor antagonist that acts to inhibit IL-1α and IL-1β binding to IL-1R preventing their inflammatory activities. Although the role of IL-1Ra has not been established in AMD pathogenesis, several studies have linked the expression of IL-1Ra to RPE cells. In human RPE cells, intracellular and secreted IL-1Ra was detected in both unstimulated and IL-1β-stimulated RPE cell cultures (131). In another study, late passage human RPE cells had an increased level of IL-1Ra compared to early passage cultures, possibly to prevent inflammation whilst under additional stress in culture (132). These authors also found that older (aged 70+) donor eyes had an increased expression of IL-1Ra in the RPE cells compared to younger donor eyes (132). Further, in mouse cultured RPE cells, inhibiting IL-1Ra expression led to a failure to suppress mature dendritic cell activation (133). These studies investigating an anti-inflammatory role for IL-1Ra expressed by RPE cells could have implications for AMD, where RPE dysfunction is a crucial mechanism for onset of retinal degeneration.

## Diabetic Retinopathy

### DR Disease Pathogenesis

Diabetic retinopathy (DR) is a major complication of diabetes, with loss of vision that arises due to unregulated high blood sugar levels, causing damage to the blood vessels and in most cases results in diabetic macular edema (DME) due to the breakdown of the blood-retinal barrier (BRB) and fluid leakage. DR can progress into two forms; non-proliferative, in which blood vessels leak or become blocked, forming microaneurysms that result in oxygen starvation to areas of the retina; or proliferative diabetic retinopathy (PDR), a more severe form characterized by neovascularisation, retinal scar tissue, and retinal detachment leading to blindness [reviewed in Duh et al. (134)]. While treatment options exist, injections of corticosteroids to reduce neovascularisation and edema are frequent and not without side effects (135). Inflammation has been implicated in the pathogenesis of DR, with increased leukocyte levels and adhesions to the vasculature, activated microglia and increased cytokine levels subsequently resulting in compromised and leaky blood vessels [reviewed in Altmann and Schmidt (68) and Tang and Kern (136)].

### Inflammasome Activation in DR

Inflammasome-mediated activation and consequent secretion of IL-1β and IL-18 is thought to play a major role in DR disease pathogenesis, with gene and protein levels of inflammasome components NLRP3, CASP1, ASC, IL-1β, and IL-18 all elevated in the peripheral blood mononuclear cell population in both non-proliferative and PDR patients, compared to controls (137). The role of the inflammasome in the pathogenesis of DR has also been investigated in rodent models of DR, and is highlighted via the use of methylene blue in streptozotocin (STZ)-induced diabetic rats, demonstrating following treatment, NLRP3 inflammasome activation including levels of IL-1β and IL-18 was reduced along with an increase in the thickness of retinal layers, and reduced permeability of the BRB (138). Additionally, in diabetic rat retinas treated with lentiviral vectors encoding *Nlrp3* short hairpin RNA (shRNA), there was a significant reduction of inflammasome components (CASP1, IL-1β, and IL-18), which correlated to a decrease in vasculature permeability when compared to diabetic controls (138). This effect was also shown following induced hyperglycaemia in diabetic rats with and without minocycline treatment, a tetracycline antibiotic, along with vascular permeability and retinal vascular apoptosis following treatment (139). Protein expression of IL-1β and IL-18 were also found to be increased in the vitreous of DR patients (137). While this finding does contrast with other reports showing that there was no change in IL-1β protein levels in the vitreous of PDR patients compared to controls, they did report increased levels of CASP1 and IL-18 in the vitreous, supporting a role of inflammasome-mediated cytokine release in DR (140). Increased levels of VEGF were also found in this study, supporting an angiogenic role of IL-18 as seen in many other retinal degenerative diseases (141).

### Angiogenic Role of IL-18 in DR

In further support of the angiogenic role of IL-18 in DR, PDR eyes with the highest levels of fulminant neovessel formation also had higher levels of IL-18 than inactive neovessel controls (141). Furthermore, CASP1 levels were reduced following anti-VEGF treatment (bevacizumab) (141). IL-18 was significantly upregulated in the serum from patients with Diabetes Mellitus Type 2 with background retinopathy, with the serum inducing a higher rate of neovascularisation when injected intradermally to mouse skin samples (mouse cutaneous angiogenesis test) compared to control serum (142). Higher serum levels of IL-18 have also been reported in Type 1 diabetic patients, half of which had a form of DR (143). While no analysis between patients with and without retinopathy was investigated in this study, the authors do remark that there was no association found between IL-18 levels and microvascular changes, however further investigation is required (143). Overall evidence suggests that IL-18 plays an important role in neovascular changes characteristic of DR, and may therefore represent a valuable therapeutic target.

### IL-1β in Cell Death in DR

The role of IL-1β in DR progression has also been widely investigated, with IL-1β protein levels found to be significantly increased in the vitreous (24, 140, 144, 145), as well as in aqueous humor (146) of DR patients. However, while IL-1β levels were shown to be significantly increased in serum from PDR patients (145), other studies have shown no change (14, 147), with this discrepancy possibly due to the different levels of breakdown of the BRB.

While systemic IL-1β inhibition using canakinumab in patients with PDR did not have an effect on neovascularisation (148), a non-statistically significant reduction in edema was evident in DME patients (148). In support of IL-1β inhibition potentially leading to more efficacy in DME than in PDR, it has been found that patients with DME have an IL-1Ra/IL-1β ratio that is 13 times higher than in PDR patients (149).

Rodent studies investigating the role of IL-1β on DR pathogenesis have also shown IL-1β to be upregulated following STZ-induced diabetes, and significantly reduced following IL-1β inhibition by anti-inflammatory cyclosporin-A administration (150), as well as following a multiple anti-oxidant diet (151) and pituitary adenylase cyclase activating peptide (152). IL-1β was also upregulated in isolated retinal vessels, compared to control rats, as well as in bovine retinal vascular endothelial cells (BREC) (153). It has also been demonstrated that following intravitreal injection of IL-1β, along with increased TUNEL-positive capillary cells in retinal microvessels, the formation of acellular capillaries had increased two-fold, both characteristic early features seen in DR pathology (151). Taken together, human and rodent models of DR suggest a role for IL-1β in cell death in this disease.

### Role of IL-1Ra in DR

The role of IL-1Ra in the progression of DR is largely unknown, however the few studies that have investigated its role largely support an anti-inflammatory or protective role. A study investigating the risk factors for the development of DR in Type 2 diabetes patients found that IL-1Ra levels in serum were negatively correlated with disease presence, with low serum levels of IL-1Ra hypothesized to be a risk marker for DR progression (27). In another study, IL-1Ra levels were found to be significantly increased in the tears of diabetic patients without retinopathy compared to those with retinopathy (42), and reduced in the plasma of diabetic patients compared to controls (43). These lower levels of IL-1Ra in diabetic patients without DR could indicate a heightened risk for developing DR, as it has been suggested that increased IL-1Ra production in diabetes could be a compensatory response to the heightened auto-immune state in diabetic patients (42). Taken together, these studies indicate a protective mechanism for IL-1Ra in preventing DR onset, as low levels of IL-1Ra could suggest that inflammation may propagate due to increased IL-1β activity.

In patients with DR however, IL-1Ra expression patterns appear to be less clear, with IL-1Ra levels along with IL-1α shown to be unchanged in the serum compared to controls (129, 132). Furthermore, following intravitreal injection of anti-VEGF agent bevacizumab in 8 patients with DR, IL-1Ra along with several other cytokines were found to be significantly lower in the vitreous than in controls (154). It is possible that as VEGF inhibition reduced neovascularisation and inflammation, IL-1Ra upregulation was not required. In addition, using the STZ-induced diabetes rat model, retinas exposed to hyperglycaemia showed significantly increased levels of IL-1ra as well as IL-1β, and their transmembrane receptors IL-1r type 1 and IL-1r type II, compared to controls (28), along with major changes in retinal architecture including compromised BRB integrity, and thinning of the ganglion cell layers (28). Evidence from studies investigating IL-1Ra levels in DR patients and rodent models could suggest an overburdening of compensatory IL-1Ra antagonist activities in more severe inflammatory states, highlighting that IL-1Ra could be a therapeutic target to prevent IL-1β and IL-18 propagation. Further investigations are still however necessary to fully elucidate the role of IL-1Ra in DR pathogenesis.

### Potential Pro-angiogenic Role of IL-37 in DR

IL-37, an anti-inflammatory cytokine in the IL-1 family, is known to inhibit the innate immune system in several models of inflammation, including hepatitis, colitis, and psoriasis (155–157). In the retina, Zhao et al. have shown that IL-37 is involved in the pathogenesis of PDR, with IL-37 levels elevated in PDR patients (47). This was also correlated with an induction of VEGF-A and pro-angiogenic cytokine angiopoietin (Ang2), indicating a potential role for IL-37 in neovascular retinal conditions (47). The authors of this study report that following IL-37 treatment in a monkey chorioretinal vessel endothelial cell line (RF/6A), tube formation and branching points were increased (85.3 and 71.4%, respectively) along with cell proliferation, compared to PBS controls (47). IL-37 has been suggested to have pro-angiogenic roles similar to IL-18 in other diseases (158, 159), able to signal through the IL-18Ra.

In another study, an upregulation of IL-37 was demonstrated in HLA-B27-associated acute anterior uveitis (AAU), inflammation of the anterior eye, which was associated with an inhibited production of a number of cytokines including IL-1β, IL-6, TNF-α, and IFN-γ (160). Further investigation into the role of IL-37 in the regulation of cytokine signaling in retinal degenerative diseases may reveal novel insights into the anti-inflammatory nature of IL-37.

## Retinitis Pigmentosa

### RP Disease Pathogenesis

Retinitis pigmentosa (RP) is an inherited form of retinal dystrophy characterized by initial rod photoreceptor degeneration, secondary cone degeneration, and retinal pigment deposits (161, 162). RP presents as a loss of peripheral vision, resulting in tunnel vision and night blindness, which in some cases ultimately progresses to full blindness (161, 162). RP has a varied etiology, including a range of non-syndromic types, as well as syndromic and systemic types, and it is caused by inherited or acquired mutations in over 50 different genes including rhodopsin (*RHO*) (163). In this disease, there is an inflammatory component to disease pathogenesis, with both increased microglial and macrophage activity (69–72) and increased levels of chemokines and cytokines found in patient and rodent models (22, 164). However, it is unclear if this increased inflammatory state is causative or a consequence of this currently untreatable disease, and the exact role that members of the IL-1 family play.

### Inflammasome-Mediated Cell Death in RP

Microglial activation can occur in both RP and late-onset retinal degeneration (L-ORD) and is a consequence of a bystander effect of rod photoreceptor cell death, causing further adjacent photoreceptor death including cones (165). Bystander photoreceptor cell death has been reported in other RP studies, including Zhao et al. that demonstrated that microglial phagocytosis of healthy photoreceptors in the retina adjacent to dying cells was evident in the rd10 mouse model of RP (70), which is a model of autosomal recessive retinitis pigmentosa where rod degeneration occurs from P18 (166). These microglia were found to express IL-1β (70). Another study demonstrated that in P23H rhodopsin mutant rats, a model of autosomal dominant retinitis pigmentosa, differential cell death pathways existed in rod and cone photoreceptors (167). It was suggested that while rod cell death occurs via heightened RIP1/RIP3/DRP1-axis mediated necroptosis, cone cell death only occurs subsequently due to bystander cell death pathways via activation of the ATP-binding P2X7 receptor and NLRP3 inflammasome activation (167). This was supported by further data that showed preserved viability of cone photoreceptors on an NLRP3-deficient mouse strain that possesses the P23H mutation (167).

Furthermore, inflammasome components were measured in three early-onset (rcd1, xlpra2, and erd) and one late-onset (xpra1) canine model of RP, with *Nlrp3, Casp1, Asc, Il-1b, Il-1ra*, and *Il-18* gene expression all upregulated in the most aggressive early-onset model, rcd1, gradually rising from the induction phase of the disease at 3 weeks and peaking in expression during the chronic cell death phase at 16 weeks (23). The expression of these inflammasome genes was also upregulated significantly in the xlpra2 early-onset model, however not until 7–16 weeks and *Il-1*β was upregulated in the late-onset model from 16 weeks. However, on examining protein expression levels, there was only a change in active IL-1β levels in the rcd1 and xpra2 models at 16 and 7 weeks, respectively. In comparison, pro-IL-18 levels were significantly reduced in both models as well as erd, with active bands not detected at all. Taken together, these results suggest an involvement of inflammasome-mediated IL-1β coinciding with photoreceptor cell death in early-onset RP (23).

This idea is supported by a study using rd10 mice, showing that increased photoreceptor cell death was correlated with increased CASP1 protein expression (168). Vitreous levels in rd10 mice, as well as patients with RP, showed increased levels of IL-1β along with reduced visual fields compared to wild type and idiopathic epiretinal membrane patient controls, respectively (22, 22), while there was no change in IL-1α levels in RP patients compared to controls, indicating that IL-1β and the inflammasome may play a role in RP.

Despite strong upregulation of inflammatory genes and IL-1β in animal models and human studies with RP, to our knowledge the other members of the IL-1 family have not been studied in the progression or onset of this disease.

## Glaucoma

### Glaucoma Disease Pathogenesis

Glaucoma defines a heterogeneous group of visual disorders that arises from compression of the optic nerve due to elevated intraocular pressure (169). Glaucoma is the leading cause of blindness in the world, and currently there is no cure. Furthermore, due to the gradual onset of vision loss, many patients are unaware they have developed this disease. In addition to optic nerve damage, glaucoma is characterized by degeneration of the retinal ganglion cells (RGC) and their axons (170, 171), a layer at the front of the retina responsible for the transmission of collated visual information to the optic nerve. There are three forms of Glaucoma, open-angle, closed angle and secondary-glaucoma, with open-angle glaucoma further divided into high or low pressure forms, named primary open-angled glaucoma (POAG) and normal tension glaucoma, respectively (169). Elevated intraocular pressure (IOP) in glaucoma can be caused by impaired aqueous outflow, either anatomically obstructed in closed glaucoma, or in open glaucoma can be caused by defective trabecular meshwork (TM) including dysregulated function of tight junctions or by build-up of plaque-like materials (15). Along with genetic and environmental risk factors, as in most retinal degenerative disorders, oxidative stress, and inflammation are believed to contribute to disease pathogenesis, augmenting IOP via the infiltration of immune cells through a leaky or impaired BRB surrounding the optic nerve, which ultimately results in RGC death and axonal injury (73, 170, 172).

### IL-18 in Glaucoma

IL-1 family members have been shown to play a role in glaucoma pathogenesis, with IL-18 expression increasing with age in the ciliary body, iris and aqueous humor of DBA/2J mice, a model of pigmentary glaucoma that naturally presents with increased IOP, RGC loss, and pigmentary dispersion (41). Levels of IL-18 appeared to precede classical pathological symptoms of this disease, peaking in expression in the iris, ciliary body and aqueous humor at 6 months. The authors therefore hypothesized that IL-18 could be a marker indicating disease onset (41).

### IL-1α and IL-1β in Glaucoma

Patients with POAG have been found to have significantly increased gene levels of IL-1β in their blood and significantly increased IL-1β protein expression in the aqueous humor compared to healthy controls (21), however in tears from POAG patients was not significant from healthy controls (173). *Il-1*α and *Il-1*β mRNAs were found to be increased in the TM in glaucomatous eyes compared to controls, acting in a feedback loop to control endothelial leukocyte adhesion molecule 1 (ELAM-1), an early marker of atherosclerotic plaque that forms in glaucoma (15). Furthermore, treatment with IL-1Ra, an IL-1R antagonist, downregulated the expression of ELAM-1 (15). These studies indicate that IL-1α and IL-1β may be involved in glaucoma pathology.

In rodent models of glaucoma, IL-1β is demonstrated to cause an increase in RGC death, hypothesized to be activated via a TLR4-NLRP1/NLRP3-CASP8-axis in an acute IOP glaucoma model in mice. In both *Tlr4*^−/−^ mice and CASP8-inhibited mice, there was reduced IL-1β production and preserved RGC health (174). Using the same IOP model in both mice and rats, another group demonstrated significantly high mRNA levels for inflammasome components *Nlrp3, Casp1, Asc*, and *Il-1*β, peaking at 1 day post-insult, however suggested that this increase in inflammatory genes was primed via the P2X7 receptor (175). P2X7-inhibited and P2X7^−/−^ mice did not demonstrate the same increase in IL-1β following damage, while the use of the P2X7 agonist bzATP promoted a surge of IL-1β again at 1 day post-insult (175). The mechanism by which P2X7-mediated IL-1β secretion occurs in glaucoma has been suggested by this group and others to occur in response to stretch and swell mechanical stresses from increased IOP (175, 176).

There exists wide speculation that given pathological similarities between glaucoma and Alzheimer's disease, a gene cluster of IL-1 polymorphisms may indicate increased risk of developing glaucoma (177). To support this idea, a study showed that the IL-1α (−889C/T) polymorphism increased IL-1 gene expression, which was associated with amyloid-β deposits that are known to accumulate in RGCs in glaucoma models (178). However, independent studies into IL-1 gene cluster polymorphisms such as C/T polymorphism in the promoter region of IL-1α, IL-1α (−889) T allele, and two C/T polymorphisms in IL-1β, rs16944 (−511 C/T) and rs1143634 (+3953C/T), have reported conflicting information on POAG and normal-tension glaucoma (NTG) disease susceptibility (179–185), promoting a meta-analysis to investigate the relationship between these polymorphisms and glaucoma risk factor. From the meta-analysis, it was concluded that there was no association between these polymorphisms and POAG or NTG development (177).

## Retinopathy of Prematurity

### ROP Disease Pathogenesis

Retinopathy of prematurity (ROP) is the leading cause of severe visual impairment and blindness in infants, that arises due to premature birth and results in underdeveloped vasculature and retinal detachment [reviewed in Shah et al. (186)]. ROP has been considered to have two phases of disease; incomplete vascularisation of the retina creating a hypoxic environment, and as a consequence, leading to neovascularisation and proliferative retinopathy (187).

### IL-18 as a Regulator of Neovascularisation in ROP

Qiao et al. determined that the expression of IL-18 was reduced in a mouse model of oxygen-induced retinopathy (OIR) (188), in which supplemental oxygen induces incomplete vascularisation of the retina, indicating that IL-18 is able to regulate neovascularisation in retinal degenerations, suggesting possible repercussions in other neovascular retinal diseases such as ROP. In humans, the development of ROP was correlated with an early decline in systemic IL-18 levels, but in later periods, correlated with increasing IL-18 levels in whole blood from 877 ROP patients (38). Incomplete retinal vascularisation during the first phase of ROP may be linked to these changes in IL-18.

### IL-1β and Choroidal Toxicity in ROP

In mouse models of OIR, IL-1β has been shown to be associated with choroidal involution, a characteristic feature of ROP (39). In this study, IL-1β was found to be increased in both the RPE and choroid, inducing toxicity in the choroid and leading to retinal and choroidal degeneration. These effects were ameliorated following IL-1β inhibition through administration of an IL-1R antagonist (39). Additionally, in a pre-term birth mouse model that induces chorioamnionitis, IL-1β was injected between the two fetal membranes on day 11 of gestation, and following birth, retinas were shown to exhibit high levels of pro-inflammatory genes accompanied by a persistent infiltration of mononuclear phagocytes in the retina (40). This was accompanied by thinning of the choroid and underdevelopment of retinal vessels. Upon antenatal administration of a non-competitive IL-1R agonist, these effects were prevented, highlighting a novel antenatal role of IL-1β on retinal vascular development (40).

In humans, levels of IL-1β were found to be unchanged and were below detectable levels in a multiplex bead cytokine array of vitreous samples from ROP and control patients (189). Further investigation into the expression levels of IL-1β is warranted, especially in the RPE and choroid.

### Role of IL-1Ra in ROP

Few studies have investigated the role of IL-1Ra in ROP pathogenesis, however this competitive antagonist was found in significantly high levels in the vitreous and tears of ROP babies, along with increased levels of VEGF, complement component proteins, and matrix metalloproteinase 9 (MMP9) (44). Furthermore, there was an increase in activated microglia/macrophages in the vitreous from ROP babies (44). As ROP is characterized by abnormal retinal vasculature development and inflammation, it is possible that IL-1Ra levels were increased in these patients as a compensatory mechanism to prevent IL-18 angiogenic effects and IL-1β-induced cell death as described in other sections. Further work is necessary to understand the role and therapeutic potential of IL-1Ra in ROP.

### IL-38 as a Novel Anti-angiogenic Factor in ROP

IL-38 is the newest member of the IL-1 family, classified under the IL-36 subfamily and has been reported to have roles in inflammation propagation in diseases such as rheumatoid arthritis, psoriasis and systemic lupus erythematosus [reviewed in Xu and Huang (190)]. IL-38 however has been largely unreported in retinal degenerations. A recent study however describes a role for IL-38 in ROP, where in a mouse model of OIR, a significantly higher level of IL-38 was found in OIR mouse retinas compared to controls (48). In addition, following IL-38 local and systemic injections in these OIR mice, angiogenesis was significantly reduced in the retinas compared to controls along with pro-inflammatory cytokine IL-1β levels (48). This was subsequently demonstrated in a cell culture model, in which VEGF-treated cells administered IL-38 had slowed wound healing following a scratch test, attenuated vascular tube formation, and reduced proliferation, processes which were eliminated with the addition of anti-IL-38 (48). It is therefore possible that IL-38 administration to ROP babies could help prevent pathogenic neovascularisation and inflammation. Further investigation is necessary to elucidate whether IL-38 may play a role in other retinal degenerative diseases, particularly in neovascular retinal diseases such as wet AMD and DR.

## Other Retinal Disorders

### Stargardt Macular Dystrophy

Stargardt macular dystrophy (STGD) is a common form of inherited macular dystrophy that leads to juvenile macular degeneration caused by an inherited autosomal recessive mutation in the *ABCA4* gene. STGD affects 1:10,000 adults and children and is characterized by progressive central vision loss resulting from lesion development in the macular region of the retina [reviewed in Tanna et al. (191) and Fujinami et al. (192)]. Although little is known about the IL-1 family members and STGD pathology, the involvement of microglia has been characterized by Kohno et al. in a *Abca4*/*Rdh8* double knockout mouse model, where activation of microglia occurred through the TLR4 signaling pathway (72), and in the same model expressed the chemokine CCL3 (193), a macrophage-inflammatory protein known to be involved in the progression of retinal degeneration (92, 194, 195). Further investigations into the role of IL-1 family members in STGD may elucidate novel inflammatory mechanisms at play during retinal degeneration in this disease.

### Retinal Vein Occlusion

Branch and central retinal vein occlusion (RVO) occurs when there is abnormal arteriovenous (A/V) crossing with vein compression and obstruction, causing degenerative changes in the vessel wall [reviewed in Laouri et al. (196) and Rehak and Rehak (197)]. Inflammation is involved in the pathology of RVO, with microglial activation and macrophage recruitment associated with an increase in pro-inflammatory cytokine production in an experimental branch RVO model (198), as well as increased levels of chemokines and cytokines including CCL2 and IL-6 in the vitreous of patients with branch RVO and macular edema (199). IL-1 family members have also been thought to play a role in disease pathogenesis in human RVO patients with retinal ischemia and recurrent macular edema, where IL-1α was significantly elevated in the aqueous humor (200), similar to in AMD patient serum (12) and in the plasma of rat ischemia/reperfusion injury (13). It has been found that RVO patients also have an increase in vitreal levels of IL-1β (19, 201), however was not elevated in the aqueous humor (202). The role of other IL-1 family members in RVO is yet to be explored.

### Retinal Detachment

A retinal detachment is a break between the neurosensory retina and the RPE, leading to fluid accumulation under the retina and sudden vision loss in the rhegmatogenous form [reviewed in Ghazi and Green (203)]. Retinal detachment can occur as a symptom of other retinal degenerative diseases including DR (134). Without prompt reattachment, retinal detachments can lead to starvation of the photoreceptors due to separation from their choroidal oxygen supply, resulting in photoreceptor cell death. Although inflammation (204), microglial migration (205), and monocyte infiltration (206) has been thought to play a role in retinal detachment, novel findings suggest that microglia may actually mediate photoreceptor cell death following retinal detachment, potentially by phagocytosing cell debris that may cause retinal damage (207). In patients with retinal detachments, elevated levels of IL-1β have been detected in the vitreous or retina (109, 208, 209), indicating a role for IL-1β in disease pathogenesis. In support of this, a study involving a mouse retinal detachment model showed that photoreceptor cell death was reduced when IL-1β and CASP1 were inhibited, as well as in *Nlrp3*^−/−^ mice with retinal detachment (109), also indicating a role for inflammasome activation in this disease. The role of other IL-1 family members in retinal detachment require further investigation.

### Autoimmune Uveoretinitis

Experimental autoimmune uveoretinitis (EAU) is a T cell-mediated autoimmune disease that is used as a model for human posterior segment uveitis, including sympathetic ophthalmia, birdshot chorioretinopathy, Vogt-Koyanagi-Harada disease, and Behçet's disease (210). Rodent EAU is induced by immunization with uveitogenic retinal proteins including the retinal soluble antigen (S-Ag) and the interphotoreceptor retinoid-binding protein (IRBP) (211). Mononuclear phagocytes have been identified to play a role in EAU, with microglial migration evident in the earlier phases of EAU and subsequent macrophage recruitment in the later phases (212). Several IL-1 family members have also been linked to the development of EAU, including IL-33 and IL-1β (213, 214), with the role of other IL-1 family members generally unknown in this disease. The expression of IL-33 was elevated in the inner nuclear layer of EAU mice compared to naïve mice (213). Interestingly, administration of IL-33 led to a decrease in EAU severity in wild type mice, alongside a reduction in T cells, IFN-γ, and IL-17 production (213), indicating that IL-33 induced a protective effect against the adaptive immune system despite its classical role as an inducer of T cell activation (127).

IL-1β has also been found to increase the severity of EAU, with the systemic delivery of recombinant IL-1β elevating EAU symptoms when administered during the priming phase of the immune response in EAU, and a decrease in EAU severity when a neutralizing antibody for IL-1β was delivered (214). IL-1β levels were found to be significantly elevated in the aqueous humor and supernatants of posterior eyecups from EAU rats (215), indicating increased production and dysfunction of IL-1β, which has been shown to cause BRB breakdown by opening the retinal vascular endothelial tight junctions in EAU (216). Another study reported that IL-1β was secreted by neutrophils, macrophages and dendritic cells in an EAU model (217). In this study, IL-1R-deficient mice had reduced severity of EAU alongside a reduction in immune cell recruitment into the retina (217), supporting other studies describing the protective effect of IL-1β neutralization in EAU (214).

## Other IL-1 Family Members in the eye

The role of other IL-1 family members in retinal degenerations remains elusive, with agonists IL-36 (α, β, and γ) and receptor antagonist IL-36Ra not being investigated in the retina, to our knowledge. Although many members of the IL-1 family have not been investigated in the retina, in patients with HLA-B27-associated AAU, changes in IL-1 family members were detected in the aqueous humor (218). Significantly higher levels of several IL-1 family members, including IL-1β, IL-18, IL-1Ra, IL-36Ra, and IL-37 was observed in AAU patient aqueous humor compared to controls (218). This study indicates that other IL-1 family members including IL-36Ra may also contribute toward ocular inflammation and may play a role in retinal degenerative diseases. In support of this, IL-36Ra has been thought to play a role in *Pseudomonas aeruginosa* keratitis, a severe corneal ulceration, with its downregulation leading to an increased severity of disease (219).

IL-36 (α, β, and γ) and IL-36Ra have been shown to play a role in the pathogenesis of other inflammatory diseases [reviewed in Ding et al. (220) and Walsh and Fallon (221)]. IL-36 cytokines have been well characterized in psoriasis, a chronic inflammatory skin condition, where the three IL-36 agonist ligands (α, β, and γ) were found to be upregulated in skin lesions [reviewed in Towne and Sims (222)]. Subsequently, a mouse model of psoriasis was created using an overexpression of IL-36α (223). IL-36 activity (IL-36 α, β, γ, or IL-36Ra) has also been linked to the pathogenesis of several autoimmune conditions, including colitis (224, 225), systemic lupis erythematosus (226), Primary Sjögren's syndrome (227) and psoriatic and rheumatoid arthritis (228, 229). Autoantibody production has also been associated with retinal degenerations such as autoimmune retinopathy (AIR) and AMD [reviewed in Morohoshi et al. (230)], and so it is possible that the IL-36 signaling axis could play a role. Several other mechanisms of IL-36 activity may also be relevant to retinal degenerative diseases; for example, it has been found that the IL-36 receptor (IL-36R) is constitutively expressed by several types of immune cells, including macrophages (231), and that IL-36α may also be expressed by macrophages (225). IL-36 agonist ligands have been shown to stimulate the production of chemokines (224) and cytokines including IL-18 (232) and IL-6 (229), also heavily involved in retinal degenerative diseases. A study showed that after stimulation with IL-1β, IL-36α, IL-36β, or IL-36γ, there was an overlap between differentially expressed genes in epidermal keratinocytes, including cytokine and chemokine production and leukocyte recruitment genes (233). The study also indicated a role for the MyD88 adaptor protein in shared IL-1β/IL-36 responses (233).

In the CNS, neuronal and glial cells have been shown to express IL-36β (234), with microglia and astrocytes thought to express IL-36R (235). However, in an experimental autoimmune encephalomyelitis (EAE) mouse model, although it was demonstrated that IL-36γ was expressed by neutrophils leading to microglial activation, IL-36γ or IL-36R deficiency did not change the severity of EAE compared to wild type controls (236). This indicates that the role of the IL-36 subfamily members in CNS diseases is unclear, and further investigation is required to determine whether IL-36 (α, β, and γ) and IL-36Ra are expressed by the retina, and if they play a role in retinal disease pathogenesis.

## IL-1β Mechanisms of Action in Retinal Degenerations

IL-1β has been the most widely studied IL-1 family member in retinal degenerative diseases, due to its broad range of pro-inflammatory functions. However, several important questions surrounding IL-1β in retinal degenerations, particularly in AMD, remain unclear; (1) IL-1β as a potential biomarker of retinal disease; (2) which inflammatory pathways it mediates; (3) which retinal cell types produce, express or secrete IL-1β; and (4) as IL-1β has no N-terminal secretory signal (237), how this unconventionally secreted protein is released from its producing cell. Therefore, this section of the review aims to summarize the current literature surrounding these themes and highlight gaps in our knowledge surrounding the role of IL-1β, particularly in the context of dry AMD.

### IL-1β as a Biomarker for Diagnosis of Retinal Degenerations

The analysis of pro-inflammatory cytokine IL-1β as a diagnostic biomarker and therapeutic target have been investigated in both ocular tissues and fluids, as well as in serum from patients with retinal degenerative diseases. Pro- and active- forms of IL-1β have been found to be upregulated in the vitreous humor (19, 22, 24–26, 109, 140, 145, 149), aqueous humor (21, 238), retina (209), and serum (12, 145, 239) of patients with retinal degenerations such as wet AMD (12, 19, 239), diabetic macular edema (149, 238), retinal detachment (109, 208, 209), RVO (19, 201), glaucoma (21), retinitis pigmentosa (22), and diabetic retinopathy (19, 24–26, 140, 144, 145, 240). However, very few studies have reported IL-1β expression levels in intraocular fluid, serum or retinal tissue in human patients with dry AMD, with reports of no significant change in IL-1β levels in AMD (mostly dry AMD patients), retinitis pigmentosa, and glaucoma, using a multiplex immunoassay system (164). In another study, no significant increase in *IL-1*β gene expression was found in the RPE of patients with geographic atrophy (GA) (103), and a non-significant increase in IL-1β levels in the aqueous humor of dry AMD patients compared to healthy controls (241). In a retrospective case-controlled study of polymorphisms in interleukin genes of nearly 500 late-stage Taiwanese dry AMD patients and controls, no single nucleotide polymorphisms (SNPs) in the IL-1β gene were found associated with the development of AMD (110), indicating little association exists between dysfunctional IL-1β gene expression and dry AMD. This suggests strongly that the dysregulation of the *IL-1*β gene might not be as important as the control mechanism which regulates its protein expression and subsequent activation through inflammasome and CASP1-mediated activation.

Further investigations into pro- and active-IL-1β levels in serum, ocular fluid, and retina in human AMD patients, particularly in dry AMD, would be of interest to determine if this pro-inflammatory cytokine may be useful as a biomarker or therapeutic target for dry AMD.

### Induction of Chemokine Production by IL-1β

Chemokines, or chemotactic cytokines, provide activation and directional cues following retinal injury to recruit immune cells to the site of damage, and are known to be regulators of leukocyte activation and recruitment in AMD (242), and have been associated with progressive retinal degeneration in mouse models of AMD (92, 129, 193, 194, 243–245). IL-1β has been implicated in the modulation of chemokine secretion via mediating NF-kB nuclear translocation allowing the genes to be subsequently transcribed (246).

Our previous work has shown that at 12 h post-injection of recombinant IL-1β into the rat eye, there was induction of retinal *Ccl2, Cxcl1*, and *Cxcl10*, key chemokines involved in leukocyte recruitment (18). This was accompanied by a significant increase in recruited macrophages into the retina through the optic nerve. Another study using ultrastructural analysis indicated that following IL-1β intravitreal injection into Lewis rats, the recruitment of mononuclear phagocytes into the retina was identified from 4 h after injection peaking at 24–48 h, accompanied by a breakdown of the BRB, edema and a higher inflammatory state (247). These studies indicate that IL-1β induction may be a mechanism by which microglia and macrophages are recruited into the damaged photoreceptor layer (18), and potentially facilitate photoreceptor cell death via phagocytosis (70). This finding is supported by a transcriptome-wide analysis of AMD retinas which showed that *Ccl2, Cxcl1, Cxcl10*, and *Cxcl11* were all upregulated in AMD retinas compared to healthy controls (248).

### The Role of IL-1β in Other Inflammatory Pathways

Several other pathways associated with AMD pathogenesis may be also affected by IL-1β production in the retina, which may lead to retinal cell death. Interleukin-6 (IL-6), a pro-inflammatory cytokine associated with pathogenesis of AMD (63, 92, 249, 250), as well as in a model of ocular toxoplasmosis (251), and has been shown *in vitro* to be regulated by IL-1β, following IL-1β-dependent activation of the p38 MAPK/NF-kB pathway (252). Regulation of NF-kB by IL-1β has also been demonstrated in a mouse model of DR following intravitreal injection of IL-1β, with concomitant increases in oxidative stress levels (8OHG and nitric oxide) and increased TUNEL-positive capillary cells, which are characteristic features of this disease (151). Adeno-associated virus (AAV) vector-mediated gene transfer of IL-1β, which was injected intravitreally, demonstrated the greatest ocular inflammatory effect on the eye even at low-dose levels, compared to AAV vectors expressing IL-6 or IL-17A (253). This led to an upregulation of inflammatory factors CXCL1, CCL2, MMP-9, VCAM-1, VEGFA, IL-6, and IL-17A, reduced photoreceptor thickness, increased cellular infiltrates, and damage to the overall structural integrity of the posterior eye (253).

Matrix metalloproteinases (MMPs), responsible for the protein degradation of the extracellular matrix (ECM) (254), have also been linked to IL-1β in retinal degenerations, with wet AMD patients carrying SNPs in *MMP-1* and *MMP-7* genes found to have a higher serum concentration of IL-1β (239). Associations between MMPs and the IL-1 family have also been found in other retinal degenerations, with increased levels of MMP-1, MMP-9, MMP-12, and IL-1β found in the vitreous of patients with POAG (21), as well as elevated MMP-9 and IL-1Ra observed in the vitreous and tears of ROP infants (44). Further, in optic-nerve induced retinal damage, increased levels of MMP-9 promoted RGC loss, which was ameliorated by an intravitreal injection of IL-1Ra (255).

Finally, the complement cascade, comprised of three pathways to trigger the lysis of pathogens, apoptotic cells and clearance of foreign debris (256, 257), may also be influenced by inflammasome signaling and may alter the level of IL-1β production in retinal degeneration. It is well established that dysregulation of the complement cascade is a critical factor in AMD pathogenesis [reviewed in Anderson et al. (258)]. Doyle et al. has demonstrated that complement component 1q (C1q), the initiator of the classical pathway, may activate the NLRP3 inflammasome in drusen using a carboxyethylpyrrole (CEP)-adducted model of dry AMD (31). We have shown that classical complement deficient (*C1qa*^−/−^) mice that had undergone photo-oxidative damage had a significant reduction in IL-1β protein expression in the progressive atrophic stages of degeneration in this model, which was associated with a reduction in inflammasome activation (259). Other complement components, including C3a (260, 261) and C5a (262), have also been thought to prime IL-1β expression by retinal cells.

### Cells Expressing IL-1β in the Retina

IL-1β has been widely reported to be expressed by cells of haematopoietic lineage (51), which in the retina encompasses resident microglia as well as infiltrating macrophages. Rodent models of retinal degenerations including dry AMD and retinitis pigmentosa, support this notion, with IL-1β localization shown to be expressed primarily by infiltrating macrophages in the outer retina and subretinal space (16, 18, 108, 109, 263). This localization pattern has also been demonstrated in non-retinal neural tissues, with IL-1β expressed in resident microglia and infiltrating macrophages of the brain following ischemic stroke (264) and in the developing cerebellum (265).

Additionally, there is scarce literature on the localization of IL-1β to any other retinal cell type in both human retinas and animal models. Recently, Chaurasia et al. localized the expression of IL-1β protein to unspecified cells in the inner retina in the Akimba mouse model of PDR (266). In other studies, intravitreal injection of NMDA induced neurotoxicity and IL-1β stimulation in Müller cells (267, 268), as well as in RGCs (268). Many studies, however, have induced the expression of IL-1β in a range of immortalized and primary retinal cell cultures lines following inflammasome stimulation (269, 270), with the majority of the literature focused on investigating the activation of the NLRP3 inflammasome in the RPE (36, 103, 104). Various *in vitro* models of retinal degenerations, using mostly RPE and microglia/macrophages in culture, have shown increased gene and/or protein expression levels of IL-1β in response to oxidative stress and inflammatory stimulations such as 4-hydroxynonenal (HNE), an end product of lipid peroxidation (271), lipofuscin components including A2E (272–275), Aβ (276–278), lysosome destabilization (104), lipopolysaccharide (LPS)-stimulated microglia-conditioned medium (279), and complement components (31, 280, 281). Taken together, these *in vitro* models, using inflammatory or oxidative stress signals characteristically found in the pathogenesis of retinal diseases, highlight potential IL-1β upregulation pathways, however lack the complexity that *in vivo* testing accounts for such as cell-to-cell interactions and retinal signaling, transport, and regulatory pathways. Furthermore, while it has been widely considered to be the predominant retinal cell type expressing the inflammasome, NLRP3 activation in the RPE has not been conclusively proven to be responsible for propagating IL-1β release and inflammatory-mediated cell death in retinal degenerations (282).

### Movement of IL-1β

There exists some discrepancy between the investigations into inflammasome activators in the RPE, and the well-reported localization of IL-1β in microglia and macrophages (16, 18, 108, 109, 263). Although it is possible and documented for the transmission of gene transcripts between the gene-producing and gene- or protein-expressing cell types (283–286), this phenomenon has not been investigated nor reported for IL-1β in the retina. However, it has been reported that extracellular vesicle encapsulation and transfer of CASP1, along with ASC and IL-1β secreted from monocytes, was able to induce a “cell death message” in vascular smooth muscle cells, a process that was inhibited using CASP1-specific inhibitor ac-YVAD-cmk (287). This process is further supported by work in pulmonary vascular endothelial cell injury showing that following LPS stimulation, active CASP1 was packaged in microparticles, along with cleaved gasdermin D, an inflammasome-dependent pyroptotic pore, and was able to stimulate endothelial cell death (288). It is therefore possible that this phenomenon could exist in the retina, with extracellular vesicle transfer of inflammasome components to microglia following receptor activation in the RPE or other host cell types. A study in which ARPE-19 cells were subject to blue-light photo-stimulation (488 nm) in culture support this hypothesis, demonstrating exosomal release with increased levels of inflammasome components CASP1, IL-1β, and IL-18 compared to unstimulated controls (289). This possibility also highlights the flaws in using only single-cell culture-based models, as it limits the ability to fully understand cell-to-cell communication and transport pathways, and prevents localization and uncovering the mechanism of how this pro-inflammatory cytokine is activated and secreted in the retina. Investigating the transport pathway of these inflammatory components using gene and protein detection methods simultaneously, as well as in the presence of gene inhibitors such as siRNA, or the use of co-culture *in vitro* systems, could shed more light on these essential cellular interactions.

## Conclusions

**Figure 1:**
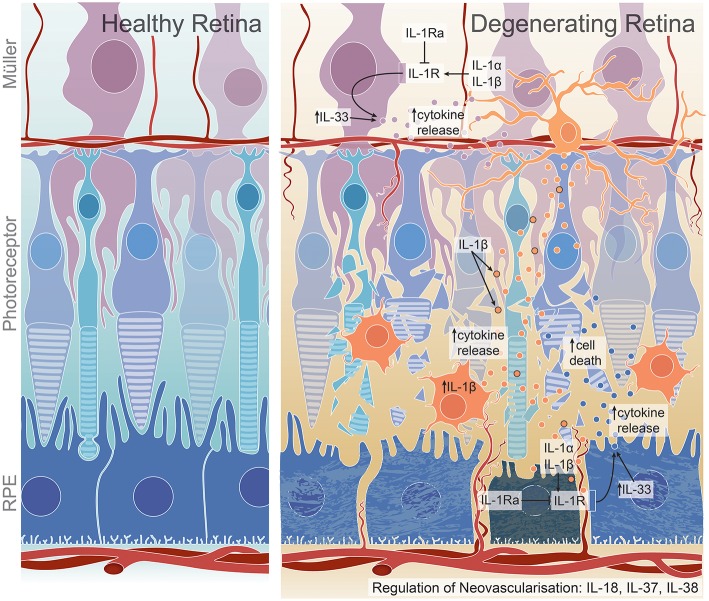
Proposed roles of IL-1 family members on cell death, inflammation, and angiogenesis in the degenerating retina. IL-1β production by activated microglia and macrophages may lead to increased chemokine and cytokine release from Müller and RPE cells, promoting further macrophage recruitment to the damaged site and ultimately resulting in photoreceptor and RPE cell death (16, 18, 108, 109). This may occur through IL-1R expression on Müller and RPE cells (18), through which IL-1α may also exert its inflammatory functions (96, 97). IL-1Ra, a competitive antagonist for IL-1R, is dysregulated in retinal degenerations (27, 44). IL-33, a less-characterized IL-1 ligand in the retina, may play a role in cytokine regulation, specifically in dry AMD pathogenesis (45, 128). IL-18, IL-37 and IL-38 all have reported roles in regulating neovascularisation; however, have been shown to have both pro- or anti- angiogenic effects, with IL-18 dysregulation conferring protection against neovascularisation in wet AMD (31), but detrimental effects in dry AMD (35), DR (141), and potentially ROP (188). Although not widely characterized, IL-37 may play a pro-angiogenic role in DR (47) while IL-38 is suggested to have anti-angiogenic roles in ROP (48).

Synergy exists between the development and progression of various retinal degenerative diseases, and the dysregulation of IL-1 family members, which contribute to either immune cell recruitment, retinal cell death, or dysfunctional angiogenesis (Figure 1). These hallmark pathogenic features are evident in both acquired and inherited forms of retinal degenerations, and are strongly correlated to the activation of the two most characterized IL-1 family members, IL-1β, and IL-18. Clear trends exist between the role of IL-1β as a regulator of cytokine production and cell death across many retinal diseases including AMD, DR, RP, glaucoma and ROP, and IL-18, which modulates neovascular aspects of these diseases. As these two pro-inflammatory cytokines are secreted in an inflammasome-dependent manner, it is well-documented that the inflammasome may play a key role in disease pathogenesis.

The role of other IL-1 family members in the retina, comprising IL-1α, IL-1Ra, IL-37, and the IL-36 subfamily (including IL-38), however, is less clear. While few studies have been performed on these members in the retinal diseases discussed in this review, taken together, evidence suggests that these cytokines may also play a regulatory role in mediating cell death, inflammation and angiogenesis in the retina. It therefore appears that the IL-1 family members may all contribute toward these major pathogenic features that typify retinal degenerations. Further investigations into the lesser-known IL-1 family members in both the retina and other neural tissues is however necessary to uncover novel mechanisms by which they may act.

While IL-1β is the most widely investigated and characterized IL-1 family member in retinal degenerative diseases including AMD, there is the limitation of testing in appropriate *in vivo* models that mimic retinal inflammasome activation, with the majority of investigative studies performed in cell culture-based systems. Although single cell culture experiments can shed light on inflammatory pathways that are active in individual retinal cell types, in order to fully elucidate the role that IL-1β plays in intercellular communication in diseases, *in vivo* testing and the use of retinal co-culture systems is necessary.

Finally, while each IL-1 family member has primarily been investigated independently of the other members, it would be worthwhile to determine how these IL-1 family members work together and how they influence each other, given the crossover between their functions in cell death, inflammation and angiogenesis. This includes IL-1Ra regulation of IL-1β, both IL-18 and IL-37 performing angiogenic functions, and a potential IL-1β/IL-36 signaling axis, briefly described in this review. Localizing IL-1 family members, as well as their receptors, will shed light on the cellular expression of these cytokines, and may elucidate novel mechanisms of action for regulating the progression of retinal degenerations.

## Author Contributions

YW, SM, RN, and NF wrote and edited the manuscript. RA-B prepared the summary figure for publication.

### Conflict of Interest Statement

The authors declare that the research was conducted in the absence of any commercial or financial relationships that could be construed as a potential conflict of interest.
